# Growth factors improve the proliferation of Jeju black pig muscle cells by regulating myogenic differentiation 1 and growth-related genes

**DOI:** 10.5713/ab.20.0585

**Published:** 2021-01-01

**Authors:** Jinryong Park, Jeongeun Lee, Ki-Duk Song, Sung-Jo Kim, Dae Cheol Kim, Sang Cheol Lee, Young June Son, Hyun Woo Choi, Kwanseob Shim

**Affiliations:** 1 Department of Animal Biotechnology, Jeonbuk National University, Jeonju 54896, Korea; 2 The Animal Molecular Genetics and Breeding Center, Jeonbuk National University, Jeonju 54896, Korea; 3 Department of Agricultural Convergence Technology, Jeonbuk National University, Jeonju 54896, Korea; 4 Division of Cosmetics and Biotechnology, Hoseo University, Asan 31499, Korea; 5 Livestock Promotion Institute, Jeju Special Self-Governing Province, Jeju 63122, Korea; 6 Cronex Inc, Cheongju 28174, Korea; 7 Department of Animal Science, Jeonbuk National University, Jeonju 54896, Korea

**Keywords:** Pig, Muscle Cell, Growth Factor, MyoD, Growth-related Gene

## Abstract

**Objective:**

The growth rate of pigs is related to differentiation and proliferation of muscle cells, which are regulated by growth factors and expression of growth-related genes. Thus, the objective of this study was to establish optimal culture conditions for Jeju black pig (JBP) muscle cells and determine the relationship of various factors involved in muscle growth with the proliferation of JBP muscle cells.

**Methods:**

Muscles were taken from the femur skeletal muscle of JBP embryos. After isolation of the muscle cells, cells were cultured in a 6-well plate under four different culture conditions to optimize culture conditions for JBP muscle cells. To analyze proliferation rate of JBP muscle cells, these muscle cells were seeded into 6-well plates at a density of 1.5×10^5^ cells per well and cultured for 3 days. Western blot and quantitative real-time polymerase chain reaction were applied to verify the myogenic differentiation 1 (MyoD) expression and growth-related gene expression in JBP muscle cells, respectively.

**Results:**

We established a muscle cell line from JBP embryos and optimized its culture conditions. These muscle cells were positive for MyoD, but not for paired box 7. The proliferation rate of these muscle cells was significantly higher in a culture medium containing bFGF and epidermal growth factor + basic fibroblast growth factor (EGF+bFGF) than that without a growth factor or containing EGF alone. Treatment with EGF and bFGF significantly induced the expression of MyoD protein, an important transcription factor in muscle cells. Moreover, we checked the changes of expression of growth-related genes in JBP muscle cells by presence or absence of growth factors. Expression level of collagen type XXI alpha 1 gene was changed only when EGF and bFGF were added together to culture media for JBP muscle cells.

**Conclusion:**

Concurrent use of EGF and bFGF increased the expression of MyoD protein, thus regulating the proliferation of JBP muscle cells and the expression of growth-related genes.

## INTRODUCTION

Pigs are economically important in the livestock industry. They are also excellent biomedical animal models [1]. Because sizes and physiological systems of pigs are similar to those of human, pigs have been used as biomedical models for human studies [2]. Jeju black pig (JBP) is one of Korean native pigs. It has been raised in Jeju Island for a long time [3,4]. Today, it is used in both the food industry and the research field. JBP has good traits such as tenderness, juiciness, and high-quality marbling that lead to an improved taste of meat [5]. However, it grows very slowly with a small body size [6].

Cultured muscle cells have been used as models to analyze muscle growth and growth mechanism by indirectly mimicking the development process of muscles *in vitro*. In addition, mass proliferation of muscle cells *in vitro* is a key requirement for the development of cultured meat, which is actively being researched as a meat alternative [7]. For primary cell culture, it is necessary to optimize cell culture conditions so that cells can proliferate and differentiate well [8]. It is also important to identify growth factors that can improve cell proliferation, activation, and viability [9]. Proper use of principal growth factors such as transforming growth factor-beta (TGF-β), insulin like growth factor 1 (IGF-1), heparin binding-epidermal growth factor (hb-EGF), and basic fibroblast growth factor (bFGF) can regulate muscle cell proliferation and myogenesis by inducing or inhibiting muscle metabolic pathways [10]. In a series of muscle regeneration processes, cytokines and growth factors can activate satellite cells, act as stimulators to induce the expression of myogenic regulatory factors (MRFs), and regulate myogenic differentiation [11].

MRFs such as MyoD, myogenin, Myf5, and MRF4 are essential transcription factors for muscle cells. These transcription factors can induce myogenesis and regulate signaling processes involved in myogenesis [12]. These MRFs have a basic helix-loop-helix domain which contains a conserved muscle recognition motif that can bind to the CANNTG site in the E-box sequence of a target gene, thus regulating the development of muscle cells [13]. Previous studies have investigated MyoD target genes using chip-seq and gene expression analysis and verified that MyoD can directly bind to many other genes expressed during muscle differentiation [14–16].

In this study, we established muscle cell lines from JBP embryos and optimized culture conditions for JBP muscle cells. We demonstrated that the expression of MyoD in JBP muscle cells was up-regulated in EGF and bFGF containing medium. Ghosh et al [1] have identified ten growth-related genes. We checked changes of these ten growth related genes in JBP muscle cells. Our results suggested that improved JBP muscle cells growth by growth factors involved the expression of those ten growth related genes at cellular level. These results can be applied to cultured meat studies in which muscle cell proliferation is a key technology *in vitro*. They can also be used as a basis for muscle regenerative medicine research.

## MATERIALS AND METHODS

### Animal care

All experimental procedures were approved by the Animal Ethics Committee of Jeonbuk National University (CBNU 2019-020), Republic of Korea.

### Cell cultures

A fetus was isolated from the amniotic membrane of a JBP at 16 and 17 weeks of pregnancy and transferred to the laboratory. Muscles were taken from the femur skeletal muscle of the pig’s hind leg thighs. Muscle samples were washed 3 to 4 times with phosphate buffered saline (PBS, Gibco, Carlsbad, CA, USA) containing 10% penicillin-streptomycin (PS, Gibco, USA). Connective tissues, blood vessels, and adipose tissues were removed. And muscle tissues were cut into small sizes. Chopped muscles were then dissociated and disaggregated with collagenase D (2 mg/mL, Roche, Indianapolis, IN, USA), dispase II (1 U/mL, Roche, USA), and 0.25% trypsin-ethylenediaminetetraacetic acid (TE, Gibco, USA) in DMEM/F12 (Gibco, USA) supplement with 10% PS at 37°C for 1 hour. After digestion, the mixture was filtered through a 70 μm cell strainer. DMEM/F12 containing 15% fetal bovine serum (FBS, Gibco, USA) was then added to the mixture to finish the digestion process. The suspension was centrifuged at 1,000×g for 5 min at 4°C and incubated with erythrocyte lysis buffer (ACK buffer) for 5 min on ice. After discarding the supernatant, cells were resuspended in culture medium (DMEM/F12) supplemented with 15% FBS and 1% PS. The cell suspension was seeded into a 100 mm cell culture dish and incubated at 37°C with 5% CO_2_ atmosphere. To separate and purify satellite cells, cell suspension was transferred to new plates 1 h later. These cells were labelled as P0 generation. Cultures were continued until cells reached about 90% confluence. Cells were then washed with PBS and dissociated with 0.25% TE for subculture on new plates.

To optimize culture conditions for JBP muscle cells, cells at passage 6 (P6) were collected and reseeded at a density of 1.5×10^5^ cells per well in a 6-well plate under the following four different culture conditions: i) culture condition I (DMEM +15% FBS+1% PS+1% L-glutamine); ii) culture condition II (DMEM+15% FBS+1% PS+1% L-glutamine+10 ng/mL EGF+10 ng/mL bFGF); iii) culture condition III (DMEM/F12+15% FBS+1% PS+1% L-glutamine); and iv) culture condition IV (DMEM/F12+15% FBS+1% PS+1% L-glutamine+10 ng/mL EGF+10 ng/mL bFGF). After selecting the culture conditions firstly, to identify which growth factors affect the proliferation of JBP muscle cells, we secondly divided into four culture conditions: i) w/o; without growth factor (control, DMEM/F12+15% FBS+1% PS+ 1% L-glutamine); ii) EGF (DMEM/F12+15% FBS+1% PS+1% L-glutamine+10 ng/mL EGF); iii) bFGF (DMEM/F12+15% FBS+1% PS+1% L-glutamine+10 ng/mL bFGF); iv) EGF+ bFGF (DMEM/F12+15% FBS+1% PS+1% L-glutamine+10 ng/mL EGF+10 ng/mL bFGF) and compared them. Every culture condition was repeated three times.

### Cell proliferation analysis

To analyze proliferation rate of JBP muscle cells, muscle cells at P6 were seeded into 6-well plates at a density of 1.5×10^5^ cells per well and cultured for 3 days. After counting the number of cells in each well, cells were reseeded at a density of 1.5×10^5^ cells per well. These processes were repeated three times until P10.

### Protein extraction and Western blotting

Total protein was extracted from JBP muscle cells. Briefly, harvested muscle cells were mixed with radio immune precipitation assay buffer (RIPA buffer, Biosesang, Sungnam, Korea) containing protease inhibitors and incubated for 40 min on ice. Cells were then centrifuged at 15,000 rpm for 30 min at 4°C to collect the supernatant. Protein concentration was determined using a DC Protein Assay Kit (Bio-Rad, Hercules, CA, USA). The same amount of protein extract was separated by sodium dodecyl sulfate–polyacrylamide gel electrophoresis using 12% gels. Separated proteins were transferred to polyvinylidene fluoride membranes. Membranes were incubated with 5% skim milk in TBST (20 mM Tris, 137 mM NaCl, 5 mM KCl, and 0.05% Tween 20) at room temperature for 1 h 30 min. They were then incubated with primary antibodies against glyceraldehyde-3-phosphate dehydrogenase (GAPDH) (1:5,000, Monoclonal, MA5-15738, Invitrogen, Carlsbad, CA, USA) and MyoD (1:1,000, polyclonal, 18943-1-AP, Proteintech, Rosemont, IL, USA) at 4°C overnight. After washing with TBST, membranes were incubated with secondary antibodies for 1 h 30 min at room temperature. Protein expression levels were detected using an ECL kit (SuperSignal WestPico Plus, Thermo Fisher, San Jose, CA, USA) and exposed with iBright CL100 Imaging System (Thermo Fisher, USA).

### Immunofluorescence

For immunocytochemistry, cells were fixed with 4% paraformaldehyde for 20 minutes at room temperature. After cells were washed with PBS, they were treated with PBS containing 3% bovine serum albumin and 0.03% Triton X-100 for 1 hour at room temperature. Cells were then incubated with primary antibodies against anti-MyoD (MyoD; polyclonal, 1:200, Proteintech, USA) and anti-paired box 7 (Pax7) (Pax7; monoclonal, 1:50, DSHB, Iowa, IA, USA). For detection of primary antibodies, fluorescently labeled (Alexa Fluor 488 or 568; Molecular Probes, Eugene, OR, USA) secondary antibodies were used according to specifications of the manufacturer.

### RNA extraction and quantitative real-time polymerase chain reaction

JBP muscle cells were collected at passages of P6 to P10. Total RNAs were extracted from these cells using an AccuZol Total RNA extraction kit (Bioneer, Daejeon, Korea). Quantity and purity of RNAs were determined using a spectrophotometer (μDrop plate, Thermo Fisher Scientific, USA). One microgram of total RNA was reverse-transcribed to cDNA with a cDNA synthesis kit (Bioneer, Korea). Quantitative real-time polymerase chain reaction (qRT-PCR) assays were performed using 1 μL of cDNA and 19 μL of stock solution containing AMPIGENE qPCR Green Mix (Enzo, San Diego, CA, USA), UltraPure distilled water (Invitrogen, USA), and primer solution containing both sense and antisense custom-designed primers on a CFX96 real-time PCR detection system (Bio-Rad, USA). Samples were denatured at 95°C for 5 minutes and cycled 40 times at 95°C (for denaturing) for 5 seconds and 60°C (for annealing and extension) for 30 seconds. Gene-specific primer sequences are listed in Table 1. The qRT-PCR results were normalized against GAPDH as a housekeeping gene to calculate the expression of each target gene.

### Statistical analysis

Statistical analysis was carried out using SAS 9.4 software program (SAS Institute Inc., Cary, NC, USA). Statistical differences were evaluated with Student’s t-test or analysis of variance (ANOVA) followed by Duncan’s multiple range test for post hoc comparisons. All data are expressed as mean± standard error.

## RESULTS

### Establishment of Jeju black pig muscle cell line *in vitro*

**Figure 1:**
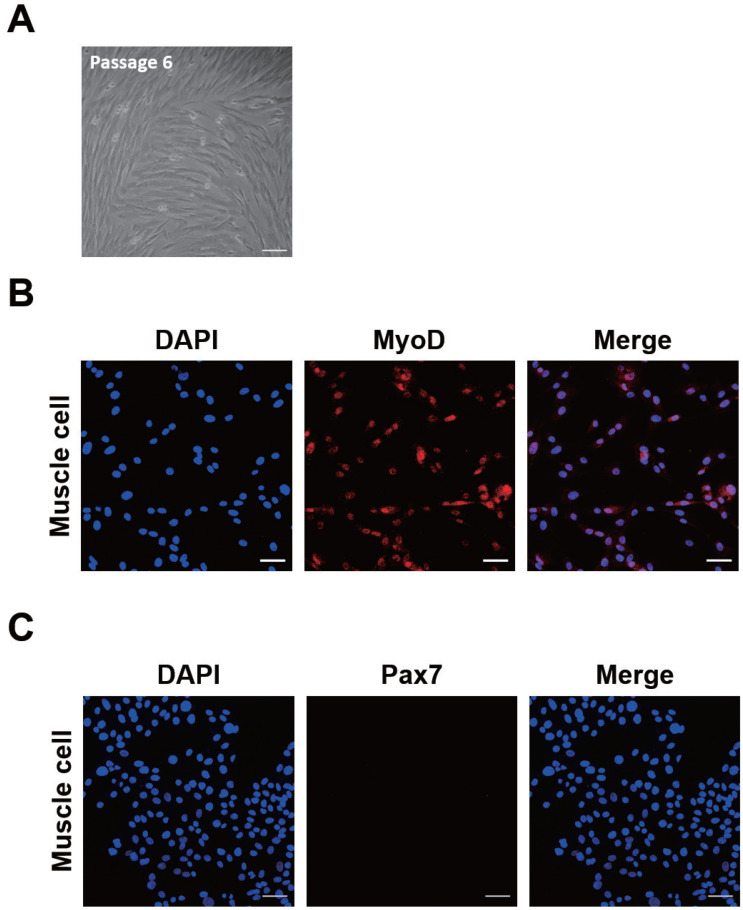
Confirmation of Jeju black pig (JBP) muscle cells. (A) Morphology of JBP muscle cells at passage 6. (B, C) Expression of myogenic markers in JBP muscle cells. Cell nuclei were stained with DAPI (blue), MyoD (red), and (green; not expressed). Scale bar, 50 μm. DAPI, 4′,6-diamidino-2-phenylindole; MyoD, myogenic differentiation 1; Pax7, paired box 7.

Muscle cells were isolated from JBP embryos and cultured in a growth medium. We tried to maintain JBP muscle cells *in vitro* for 6 passages (Figure 1A). These cultured muscle cells were positive for MyoD, but not for Pax7 (Figures 1B–C). This indicates that the cultured muscle cells are not satellite cells but are differentiated myoblasts (myocytes).

### Optimization of culture medium for Jeju black pig muscle cells

**Figure 2:**
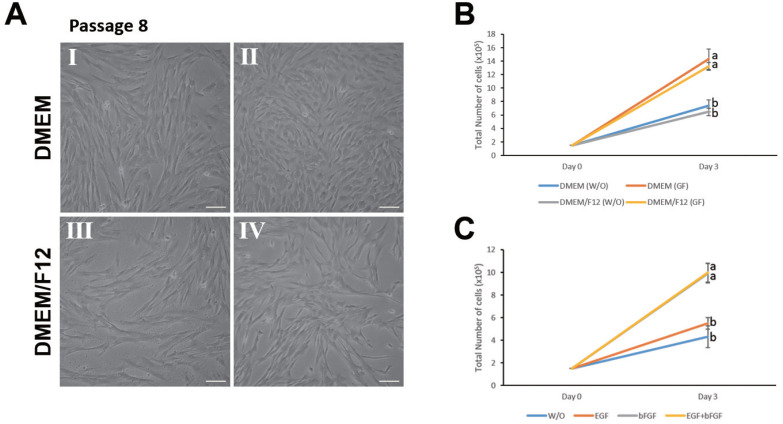
Comparison of morphology and proliferation of JBP muscle cells according to culture conditions. (A) JBP muscle cell morphology under different culture conditions using different media: I (DMEM); II (DMEM+EGF+bFGF); III (DMEM/F12); IV (DMEM/F12+EGF+bFGF). (B) Cell proliferation in culture medium containing growth factors was higher than that in culture medium without the addition of growth factors. (C) Proliferation of JBP muscle cells under various growth factor conditions. w/o (DMEM/F12); EGF (DMEM/F12+EGF); bFGF (DMEM/F12+bFGF); EGF+bFGF (DMEM/F12+EGF+bFGF). Values are presented as mean±standard error. ^a,b^ Different letters represent statistically significant differences among treatments (p<0.01). Scale bar, 50 μm.

To optimize culture conditions for JBP muscle cells, the cells were cultured in four different culture media (I, DMEM; II, DMEM+EGF+Bfgf; III, DMEM/F12; and IV, DMEM/F12+ EGF+bFGF) (Figure 2A). The morphology of muscle cells of Passage 8 under different culture conditions of I, II, III, and IV was similar to that of Passage 6 (Figures 1A, 2A). To determine culture condition for JBP muscle cells, the number of cells upon passaging in four culture mediums was determined. We found that the proliferation rate of these muscle cells in culture condition II or IV was higher than that in culture condition I or III even after repeating passages (more than 10 passages) (Figure 2B). After firstly selecting the optimal culture conditions (culture condition IV), to identify which growth factors could affect the proliferation of JBP muscle cells, growth rates of JBP muscle cells under various conditions added with different growth factors were compared. Cell proliferation was found to be higher in a culture medium containing bFGF or a culture medium containing both EGF and bFGF than that in a culture medium without adding any growth factor (w/o) or a culture medium containing EGF only (Figure 2C).

### MyoD expression induced by growth factors

**Figure 3:**
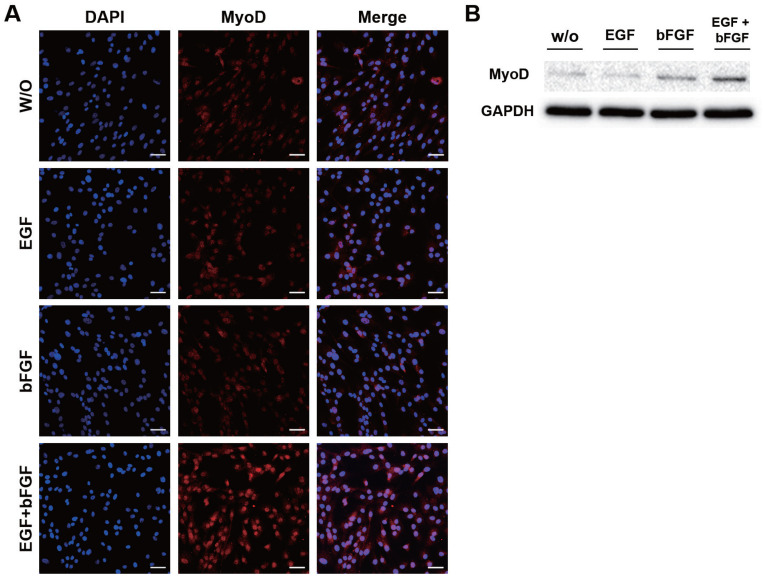
Increased MyoD expression in Jeju black pig (JBP) muscle cells when culture medium contained both EGF and bFGF. (A) MyoD was expressed in nuclei of JBP muscle cells. These cell nuclei were stained with DAPI (blue), MyoD (red). (B) Protein level of MyoD in JBP muscle cells. Scale bar, 50 μm. DAPI, 4′,6-diamidino-2-phenylindole; MyoD, myogenic differentiation 1.

The expression of MyoD in JBP muscle cells was analyzed by immunofluorescence. MyoD is a MRF that plays an important role in skeletal muscle growth and differentiation. When only EFG or bFGF was added to the culture medium of JBP muscle cells, MyoD expression was not significantly different from that in the control. However, MyoD expression in JBP muscle cells was up-regulated in cells cultured with a medium containing both EGF and bFGF (Figure 3A). Results of western blot for MyoD showed that MyoD protein level was increased in a culture condition with both EGF and bFGF (Figure 3B).

### Gene expression pattern in Jeju black pig muscle cells

**Figure 4:**
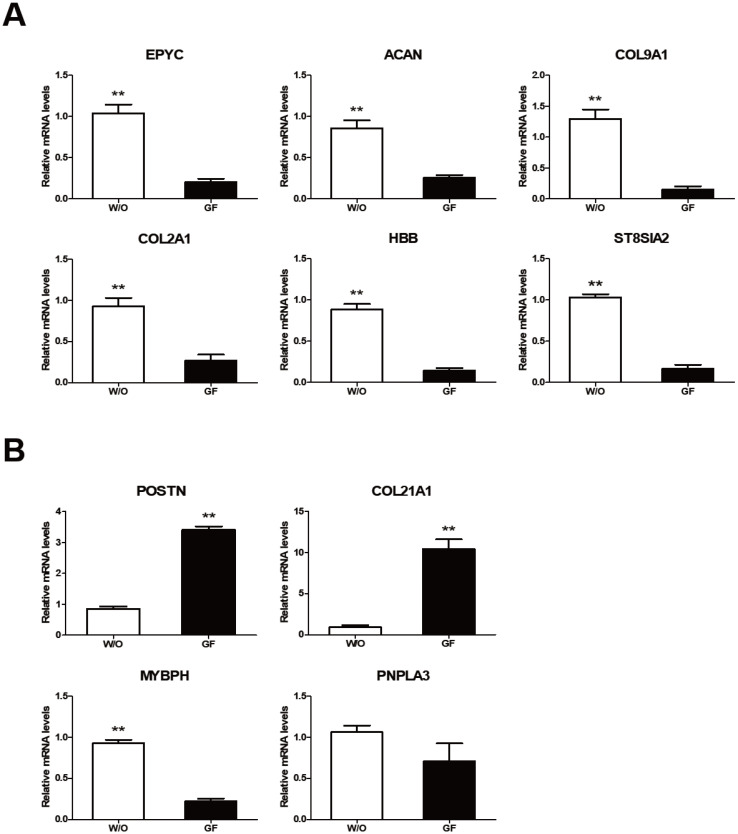
Growth-related gene expressions in Jeju black pig (JBP) muscle cells according to culture addition. (A) Down-regulated genes and (B) up-regulated genes in JBP muscle cells. w/o (DMEM/F12); GF (DMEM/F12+EGF+bFGF). Values are presented as mean±standard error. Significant differences are indicated by ** p<0.01.

We analyzed expression levels of ten growth related genes reported by Ghosh et al [1] based on their analysis of JBP muscle tissues. Ghosh et al [1] have shown that six (epiphycan [*EPYC*], aggrecan [*ACAN*], collagen type IX alpha 1 [*COL9A1*], collagen type II alpha 1 [*COL2A1*], hemoglobin subunit beta [*HBB*], and ST8 α-N-acetyl-neuraminide α-2,8-sialyltransferase 2 [*ST8SLA2*]) of these genes are down-regulated while four genes (periostin [*POSTN*], collagen type XXI alpha 1 [*COL21A1*], myosin binding protein H [*MyBPH*], and patatin like phospholipase domain-containing 3 [*PNPLA3*]) are up-regulated in JBP muscles. In our study, expression levels of these genes were different depending on culture conditions of JBP muscle cells. Expression levels of the six down-regulated genes reported by Ghosh et al [1] were significantly higher under a culture condition without growth factors (Figure 4A). On the other hand, expression levels of two up-regulated genes (*POSTN* and *COL21A1*) reported by Ghosh et al [1] were significantly higher under culture conditions containing growth factors, although *MyBPH* gene expression was significantly higher in a culture medium without growth factors (Figure 4B). *PNPLA3* gene expression was not significantly different among treatments.

**Figure 5:**
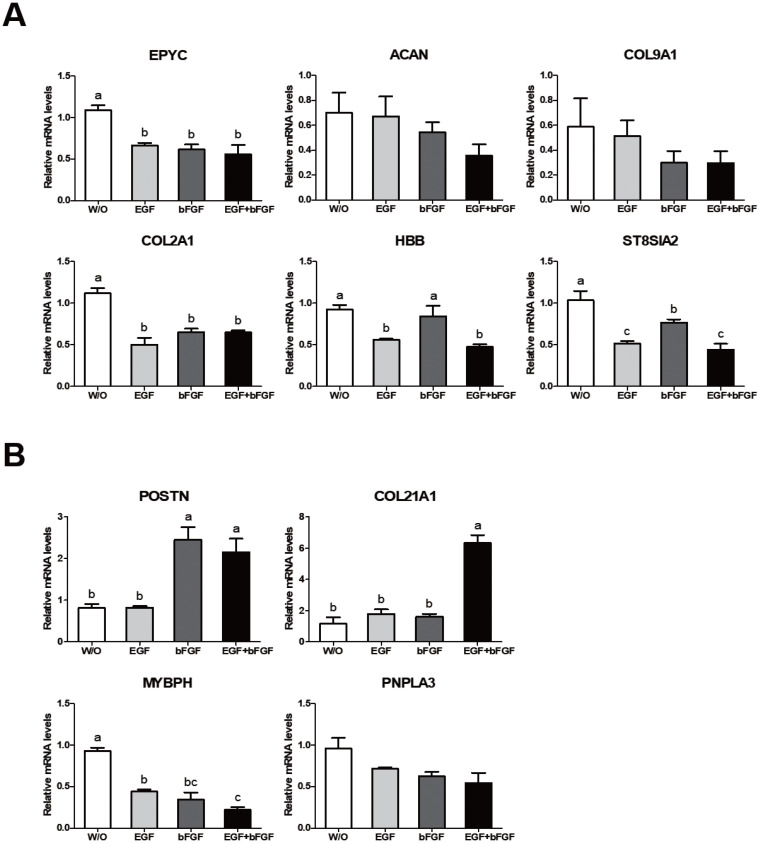
Growth-related gene expressions in Jeju black pig (JBP) muscle cells according to growth factor added to culture medium. (A) Down-regulated genes and (B) up-regulated genes in JBP muscle cells. w/o (DMEM/F12); EGF (DMEM/F12+EGF); bFGF (DMEM/F12+bFGF); EGF+bFGF (DMEM/F12+EGF+bFGF). Values are presented as mean±standard error. ^a–c^ Different letters represent statistically significant differences among treatments (p<0.01).

Gene expression patterns in cells grown in culture media containing different growth factors were different depending on the growth factor. As shown in Figure 5A, EGF was an important factor for the expression of *ST8SIA2* and *HBB* genes. In addition, expression levels of *EPYC* and *COL2A1* genes in cells cultured with a medium containing a growth factor were significantly lower than those in cells cultured in a medium without any growth factor. Their expression levels showed no significant difference between groups containing various growth factors (Figure 5A). Expression patterns of up-regulated and down-regulated genes shown in Figure 5B were similar to those shown in Figure 4B. Expression levels of *POSTN* and *COL21A1* genes were significantly higher in cells under culture conditions containing both EGF and bFGF, whereas the expression level of *MyBPH* gene was the highest in cells under a culture condition without any growth factor (Figure 5B). It was found that the expression of *COL21A1* gene was changed only when EGF and bFGF were both added to culture media for JBP muscle cells (Figure 5B). These results indicate that the expression of genes related to muscle growth is regulated by growth factors added to the cell culture medium.

## DISCUSSION

For cell culture *in vitro*, optimization of the culture medium is crucial for cell proliferation [17]. In this study, to find the optimal culture conditions for muscle cells extracted from embryos of JBPs, we compared cell proliferation under four different culture conditions. By comparing cell proliferation rates, we found that culture condition containing growth factors of both bFGF and EGF was an optimal condition for culturing JBP muscle cells. When skeletal muscles are damaged, cytokines and growth factors are released into wounded sites. Satellite cells are then activated to regenerate new muscle fibers [18]. Growth factors are known to play important roles in musculoskeletal processes and disorders such as skeletal muscle growth, fracture healing, repair of articular cartilage, and osteoporosis [19]. They can stimulate or inhibit cell proliferation and affect cell differentiation through signaling pathways of each factor [20,21]. Previous study has shown that bFGF is a potent growth factor that can stimulate the proliferation and fusion of myoblasts *in vitro* [21]. Moreover, bFGF can also induce muscle development by activating FGF receptor tyrosine kinase [22]. Furthermore, EGF can also enhance cell proliferation and the percentage of cells positive for myocyte marker [23]. In our research, the proliferation rate of muscle cells was increased when bFGF was added to culture medium alone or in combination with EGF and bFGF. These results confirm that bFGF is a more effective factor for muscle cell growth than EGF *in vitro*.

Immunostaining of JBP muscle cells revealed that MyoD expression was specifically increased by the presence of both EGF and bFGF. MyoD, MRF, is an important factor in formation and development of skeletal muscle along with myogenin, Myf5, and MRF4. As a muscle-specific transcription factor, MyoD can regulate gene expression with functions such as transcription factor activity and cell cycle regulation [13]. It can form a cross regulatory transcription network that plays a critical role in muscle cell determination and differentiation [24]. Myogenesis is regulated by the expression of MRFs [25] and the interaction of signaling substances (bFGF, Wnt, Shh, TGF-β1, and IGFs) secreted by neural tube and notochord [26]. bFGF can increase the expression of important myogenic proteins like MyHC and myogenin. It can also promote myogenesis via the PI3K-Akt-mTOR signaling pathway, thereby improving skeletal muscle regeneration [27]. Liu et al [28] have reported that both EGF and bFGF can promote muscle cell proliferation individually. However, when they are used together, they can act more quickly and potently. Our study demonstrated that EGF in combination with bFGF could induce somite myogenesis, resulting in increased MyoD expression.

Previous studies have attempted to identify genes affecting muscle development and growth. Ghosh et al [1] have selected genes related to muscle growth that are differentially expressed from muscle tissues of Jeju native pig and miniature pig. Among those genes reported by Ghosh et al [1], 6 down-regulated genes and 4 up-regulated genes in Jeju native pigs were analyzed at cellular level in the present study. In the present study, expression levels of growth-related genes were changed after growth factors were added to the culture medium. The expression pattern of each gene was different depending on the growth factor added. Nain et al [29] have reported that *ACAN*, *COL9A1*, and *COL2A1* genes are involved in cell adhesion and skeletal system development during myogenesis. In addition, ACAN is also a cartilage specific matrix protein. Along with COL2A1, its up-regulation can help Pax7 positive satellite cells differentiate along the musculoskeletal lineage to form skeletal muscle [30]. However, in our results, cell proliferation rate of cells with added growth factor was high, although expression levels of these two genes in the presence of growth factors were lower than those in the control without the addition of a growth factor.

In this study, the *HBB* gene was confirmed to be regulated by EGF. *HBB* gene encodes a subunit of adult hemoglobin [31]. Mutated HBB can cause sickle cell disease, a common human genetic disease [32]. In tissue culture, HBB has reported to be able to mediate growth arrest and apoptosis of neuroblastoma cells [33]. Similar to HBB, *ST8SIA2* gene also had a significantly lower expression when the culture medium contained EGF. ST8SIA2, also known as STX, is a type II membrane protein that is a member of the glycosyltransferase family [34]. It is an enzyme responsible for the transfer of polysialic acid to glycoproteins [35]. Up-regulated *ST8SIA2* gene expression in neural cells can cause apoptosis [36]. In contrast, cell viability can also be controlled by the expression level of *ST8SIA2* gene [37].

POSTN has been reported to be expressed in C2C12 myoblasts [38]. This expression is regulated by TGFB1 [38]. Moreover, the expression of *POSTN* gene is up-regulated by bFGF through p38MAPK signaling pathway [39]. In the present study, the expression of *POSTN* gene was high when the culture medium contained bFGF. On the other hand, COL21A1 is expressed in skeletal muscles as well as in various tissues such as the stomach and heart [40]. It can induce proliferation and migration of smooth muscle cells [41]. Nihashi et al [42] have reported differentially expressed genes in myoblasts of layer chickens compared to those in broiler chickens. In broiler chicken’s myoblasts with higher proliferation and differentiation ability than layer chicken’s myoblasts, *COL2A1* gene was down-regulated, and *COL21A1* gene was up regulated. They reported that these collagens contribute to the formation of a niche in muscle stem cells [42]. Expression patterns of these two collagen genes (*COL2A1* and *COL21A1*) in their study were similar to those found in the present study. These results suggest that *COL2A1* and *COL21A1* genes are regulated by growth factors and involved in muscle growth.

In conclusion, this study suggested optimal culture conditions for muscle cells and demonstrated that simultaneous use of EGF and bFGF could promote muscle cell proliferation *in vitro* by regulating the expression of MyoD, an important transcription factor for muscle development, and growth-related genes. Our results can be used to produce cultured meat through proliferation of muscle cells *in vitro*. These results also provide new evidence to promote the growth of pig muscles *in vivo* by analyzing the mechanism of growth and differentiation of muscle cells. However, further studies are needed on the maintenance of muscle stem cells for mass production *in vitro* and molecular regulatory mechanisms between MRFs and muscle growth related genes.

## Figures and Tables

**Table 1:** Primers used for quantitative real-time polymerase chain reaction

Gene name	Primer sequences	Accession number	Length (bp)
*GAPDH*	F: 5′-CTCAACGGGAAGCTCACTGG-3′	NM_001206359.1	280
	R: 5′-CATTGTCGTACGAGGAAATGAGC-3′		
*EPYC*	F: 5′-CCCTCTGCCTCTTCCAGAAA-3′	XM_021093029.1	199
	R: 5′-AACAGGCAAACGAGGTAGG-3′		
*ACAN*	F: 5′-CAGAAGCTGTGTGAGAACGG-3′	NM_001164652.1	176
	R: 5′-TGGTAGTCCTGAGCGTTGTT-3′		
*ST8SIA2*	F: 5′-ACCTTGAAACCCGGAGACAT-3′	NM_001315676.1	109
	R: 5′-TGTTCTTCAGCGGAGAGGTT-3′		
*COL9A1*	F: 5′-GAGCAAGTTGGCGTGAAGAT-3′	XM_003121273.4	164
	R: 5′-AGAGTTGCGCTGTTCCTTTC-3′		
*HBB*	F: 5′-GGCAAAGTGAATGTGGACGA-3′	NM_001144841.1	168
	R: 5′-GAAGGACTGGAGCACCTTCT-3′		
*COL2A1*	F: 5′-GGCTCCCAGAACATCACCTA-3′	XM_021092611.1	226
	R: 5′-GGCGAGAGGTCTTCTGTGAC-3′		
*COL21A1*	F: 5′-GAATCCGTCTGTCCAACACG-3′	XM_013977769.1	183
	R: 5′-TGGGAACACATTGCTTGTGG-3′		
*PNPLA3*	F: 5′-TCCTCCACCCATCCTTCAAC-3′	NM_001146126.1	168
	R: 5′-GGACTTCCTCTTTGGACCGA-3′		
*MYBPH*	F: 5′-ACTCAGCTCTTCTGCAGTGT-3′	NM_001033014.1	105
	R: 5′-CTCAGAGATGGCCCGGTATT-3′		
*POSTN*	F: 5′-CAAACAGCTCAGGGTCTTCG-3′	NM_001206347.1	198
	R: 5′-TCTGCAGCTTCAAGTAGGCT-3′		

*GAPDH*, glyceraldehyde-3-phosphate dehydrogenase; *EPYC*, epiphycan; *ACAN*, aggrecan; *ST8SIA2*, ST8 α-N-acetyl-neuraminide α-2,8-sialyltransferase 2; *COL9A1*, collagen type IX alpha 1; *HBB*, hemoglobin subunit beta; *COL2A1*, collagen type II Alpha 1; *COL21A1*, collagen type XXI alpha 1; *PNPLA3*, patatin like phospholipase domain-containing 3; *MYBPH*, myosin binding protein H; *POSTN*, periostin.
